# Supplementary material for: Cardiac Biomarkers in a Brazilian Indigenous Population Exposed to Arboviruses: A Cross-Sectional Study
Source: Viruses. 2024 Dec 10;16(12):1902. doi: 10.3390/v16121902 (PMC11680384; doi:10.3390/v16121902)
Supplement: Supplementary file 1 [file viruses-16-01902-s001.zip › viruses-3265505-supplementary.pdf]

Supplementary file

Atherosclerosis among Indigenous Populations (PAI): Cardiac biomarkers in the Brazilian indigenous population exposed to arboviruses: a cross-sectional study

Patient Data Collection Questionnaire

1. Patient Identification
Name: ____

Age: __________ Gender: __________

Patient ID: _______________________________________

2. Clinical Evaluation
Cardiovascular Disease

Yes [ ] No [ ]

Comorbidities

Diabetes Mellitus (DM) [ ]

Hypertension (HTN) [ ]

Chronic kidney disease dialysis [ ]

Obesity [ ]

3. Arbovirus Serological Status
Dengue: Relative Index _______

Zika: Relative Index _______

Chikungunya: Relative Index _______

Cutoff for Serological Values:

Positive: Relative Index ≥ 1.1

Borderline: Relative Index ≥ 0.8 and < 1.1

Negative: Relative Index < 0.8

5. Transthoracic Echocardiogram (TTE) Data

Ejection Fraction (%): __________

Left Ventricular Systolic Volume (ml): __________

Left Ventricular Diastolic Volume (ml): __________

TAPSE (mm): __________

Myocardial Mass (g): __________

6. Blood Pressure

Systolic/Diastolic Blood Pressure: __________ / __________ mmHg

Use of Antihypertensive Medication: Yes [ ] No [ ] If yes, specify: __________________

7. Anthropometric Data

Weight (kg): __________

Height (m): __________

Body Mass Index (BMI): __________

8. Laboratory Tests

Fasting Blood Glucose (mg/dL): __________

Hemoglobin A1c (HbA1c) (%): __________

8.1. Inflammatory Biomarkers

Cardiotrophin1 (CT-1): __________

Growth Differentiation Factor-15 (GDF-15): __________

Lactate Dehydrogenase B (LDH-B): __________

Fatty Acid Binding Protein 3 (FABP3): __________

Myoglobin: __________

N-terminal pro-brain natriuretic peptide (NT-proBNP): __________

Cardiac Troponin I (cTnI): __________

Big Endothelin-1 (Big ET-1): __________

Creatine Kinase MB (CKMB): __________
